# Sequencing of the VP1–2A Genome Segment for Hepatitis A Subtyping

**DOI:** 10.17691/stm2020.12.1.06

**Published:** 2020

**Authors:** A.A. Zalesskikh, T.N. Bystrova

**Affiliations:** Senior Researcher, Laboratory for the Epidemiology of Viral Hepatitis, Blokhina Scientific Research Institute of Epidemiology and Microbiology of Nizhny Novgorod, Russian Federal Service for Surveillance on Consumer Rights Protection and Human Wellbeing (Rospotrebnadzor), 71 Malaya Yamskaya St., Nizhny Novgorod, 603950, Russia; Professor, Leading Researcher, Laboratory for the Epidemiology of Viral Hepatitis, Blokhina Scientific Research Institute of Epidemiology and Microbiology of Nizhny Novgorod, Russian Federal Service for Surveillance on Consumer Rights Protection and Human Wellbeing (Rospotrebnadzor), 71 Malaya Yamskaya St., Nizhny Novgorod, 603950, Russia

**Keywords:** hepatitis A, genotyping, sequencing

## Abstract

**The aim of the study** was to improve the VP1–2A sequencing method and facilitate the hepatitis A virus genotyping technique.

**Materials and Methods:**

Nucleic acids were extracted by using commercial kits for nucleic acid extraction and reverse transcription in a manual and automatic version. Primer sequences were selected using the DNASTAR software. Oligonucleotides were synthesized by the amidophosphite method followed by purification with polyacrylamide gel electrophoresis. The sought fragments of the genome were obtained with PCR. Commercial components were used to prepare the reaction mixture. The results were verified by agarose gel electrophoresis with ethidium bromide DNA staining. After amplification, the DNA fragments were purified by adsorption on silica columns. Sequence identification of the purified fragments was performed by the Sanger sequencing method. The obtained sequences of the VP1–2A fragment were analyzed by the MEGA 6 software package. The optimized technique for sequencing of the VP1–2A segment was tested with 171 clinical and environmental samples containing hepatitis A virus RNA.

**Results:**

The sequencing technique has been optimized at the stage of amplification of the variable VP1–2A structural genome segment. To obtain this result, we created original primer sequences and selected the polymerase reaction conditions (length, localization and annealing temperature, concentration of the primers and template). The sought fragment with a length of 249 nucleotide pairs was located between the 2988^th^ and 3237^th^ base pairs as per the reference HM175 strain. Using this optimized method of VP1–2A fragment sequencing, the hepatitis A genotype was detected in 76 from 171 samples containing hepatitis A virus RNA in the city of Nizhny Novgorod from 2000 to 2017. The prevalence of the IA subtype was found in 97% of cases (74 of 76 samples), and only 2 samples contained subtype IB.

**Conclusion:**

The sequencing technique optimized at the stage of VP1–2A fragment amplification allows one to determine the hepatitis A virus genotype, identify imported virus strains, and also establish an epidemiological relationship between different cases of the disease.

## Introduction

Hepatitis A continues to be the most common cause of viral hepatitis. Over the two recent years, more than 25 thousand cases of this disease have been recorded in countries of Western Europe, traditionally considered as low endemic territories [1–3].

In Russia, hepatitis A is also attributed to infections of great socio-economic importance. The registered incidence rate in Russia fluctuated over the past 10 years in a sizable range of 4.29 to 37.9 cases per 100 thousand population. The number of massive hepatitis A outbreaks increased by 20% from 2016 to 2017 [4–7]. There are pronounced differences in the incidence rate between different regions: in some of them, the incidence of hepatitis A exceeded the country’s average by 1.5–4 times. In the recent decade, this viral infection has persistently ranked second after chronic hepatitis C in terms of economic losses. In 2017, it amounted to more than 1 billion rubles [5, 7–9].

Molecular genotyping is a modern tool for studying the manifestations and epidemic process of hepatitis A as well as the mechanisms of its regulation. Molecular genetic technologies allow one to study the pathogen variability and significantly increase the accuracy of diagnosis, especially in epidemic foci. Such factors as the source of infection, transmission pathways and the epidemiological relationship between infected individuals are now analyzed using these methods. In Russia, however, there are few studies on the genotypic structure of the hepatitis A virus circulating in various territories of the country. Research is difficult due to the lack of commercial reagent kits for genotyping.

The genetic variation of the pathogen is most often studied using the sequencing method, for which the amplification of a genome fragment is performed. To run the reaction, original or reported in the literature primers sequences are used; the performance of these different assays varies greatly [10–15].

**The aim of the study** was to improve the VP1–2A segment sequencing method and facilitate the hepatitis A virus genotyping technique.

## Materials and Methods

Nucleic acid extraction and RNA reverse transcription into complementary DNA were performed using commercial kits for nucleic acid isolation and reverse transcription in a manual or automatic version using a robotic station (Xiril, Switzerland).

The selection of primer sequences was performed using the PrimerSelect software (DNASTAR, USA). Oligonucleotides were synthesized by the amidophosphite method, followed by purification with polyacrylamide gel electrophoresis (Syntol, Moscow).

Commercial components were used to prepare PCR (Interlabservice, Russia): PCR buffer (MgCl_2_ — 15 μmol/L), a mixture of deoxynucleoside triphosphates (1.74 μmol/L each), and Taq polymerase (5 units/μl). The amplification reaction was run with the help of a Maxygene Therm-1000 thermocycler (Axygen, USA).

Electrophoresis of PCR products was performed on 1.5% agarose gel with ethidium bromide DNA staining; the results were recorded using the gel documentation system G:Box F 3 (Syngene, USA).

After amplification, the DNA fragments were purified by adsorption on silica columns supplemented with PCR purification reagents (Qiagen, USA). The sequence of the purified fragments was determined using an ABI PRISM 3100 genetic sequencer (Applied Biosystems, USA) according to the manufacturer’s instructions.

The obtained sequence of the VP1–2 A fragment was analyzed by the MEGA 6 software, as well as using the online BLAST services (NCBI, USA) and the Hepatitis A virus genotyping tool (RIVM, Netherlands, http://www.rivm.nl/mpf/typingtool/hav/introduction).

The optimized sequencing technique for the VP1–2A region was tested in 171 clinical and environmental samples containing hepatitis A virus RNA. For the study, clinical material from hepatitis A patients and wastewater concentrates collected in Nizhny Novgorod in 2000–2017 were used.

## Results and Discussion

As a target for sequencing, the VP1–2A fragment of the structural part of the hepatitis A virus genome was selected; this fragment has a fairly high variability compared to other regions of the genome. The NCBI Genbank library has accumulated a large number of virus isolates from various countries, which have been characterized by this specific region [16, 17].

To optimize the sequencing techniques at the phase of amplification of the VP1–2A fragment, we used our original primers with sequences described by Robertson et al. in their pioneer studies on hepatitis A virus genotypes [18]. Our modification included bringing the melting point of the oligonucleotides closer to each other in order to minimize the likelihood of forming hairpins or primer dimers. The optimal parameters were reached by changing the primers lengths and location of their annealing using the DNASTAR software, which significantly increased the amplification performance as compared to the prototype oligonucleotides. The sequence of the primers is shown in the Table.

**Table:** Primers for amplification of the VP1-2A fragment of the hepatitis A virus genome

Type of primer	Sequence	Length of nucleotides	Localization*
Forward	5`-AGAGCTCCATTGAACTCA-AATGC-3`	23	2988–3010
Reverse	5`-AAAACAGTCCCTTCATTTTCCTAGG-3`	25	3213–3237

* Nucleotide numbering is given according to the reference HM175 strain.

The length of the amplified fragment of interest was 249 nucleotide base pairs (see the Figure). The reaction mixture included 5 μl of PCR buffer, 2.5 μl of a deoxynucleoside triphosphate mixture, 2.5 μl of the forward and reverse primers (3 μmol/L, each), 0.25 μl of Taq polymerase, and 2.25 μl of deionized water. The total volume of the reagent mixture was 25 μl including 10 μl of complementary DNA of the test sample.

**Figure:**
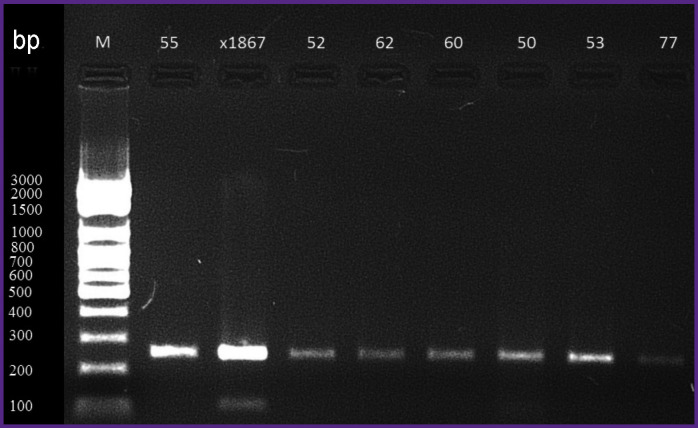
Electrophoresis of amplified VP1–2A fragments of the hepatitis A virus genome with a length of 249 nucleotides (bp); the left row is a molecular weight marker

For the amplification phase, the following thermal cycler program was used: 95°С — 5 min, then 42 cycles (95°С — 10 s, 53.5°С — 10 s, 72°С — 10 s), and the final elongation at 72°C — 1 min. The primer annealing temperature (53.5°C) was selected so to achieve the maximum yield of the reaction product that was then quantified by electrophoresis.

The variable VP1–2A fragment of the hepatitis A virus genome was detected in 76 samples out of 171 positive for hepatitis A virus RNA. This result can be associated with a low concentration of RNA in the samples and differences in sensitivity between the RNA test and the described sequencing procedure, which requires further investigation.

After amplification, the samples were purified from by-products, and then the sequence of the obtained fragments was determined using an automatic sequencer. The obtained VP1–2A sequences were equalized with each other and analyzed for homology with hepatitis A virus isolates of various geographical origins deposited in Genbank; the MEGA 6 software package was used for this data analysis.

The proposed technique was used for the first time to discover the predominant circulation of the subtype IA genotype of hepatitis A virus among the population of Nizhny Novgorod in 2000–2017. The IA subtype was found in 74 out of 76 samples, and only 2 samples contained the subtype IB virus.

Phylogenetic analysis showed that the subtype IA isolates were 93% homologous to the sequences isolated in the European part of Russia, which formed a single phylogenetic cluster. This is consistent with reports on hepatitis A virus genomic variants circulating in the European part of Russia [13, 19]. The identified subtype IB strains had maximum homology with isolates from Egypt and Bulgaria, which indicated probable imported cases of hepatitis A infection [20].

## Conclusion

The optimized amplification stage of the sequencing technique for the VP1–2A fragment of the hepatitis A virus genome made it possible, for the first time, to determine the genotypic structure of circulating strains of this virus in Nizhny Novgorod in 2000–2017. The proposed technique can be used to monitor the circulation and variability of the hepatitis A virus, investigate multiple cases of infection, establish an epidemiological relationship between different cases, and identify imported hepatitis A virus strains.
